# Relationships between Airway Remodeling and Clinical Characteristics in COPD Patients

**DOI:** 10.3390/biomedicines10081992

**Published:** 2022-08-17

**Authors:** Andrew Higham, Josiah Dungwa, Natalie Jackson, Dave Singh

**Affiliations:** 1 Division of Immunology, Immunity to Infection and Respiratory Medicine, School of Biological Sciences, Faculty of Biology, Medicine and Health, University of Manchester and Manchester University NHS Foundation Trust, Manchester M23 9LT, UK; 2 Medicines Evaluation Unit, The Langley Building, Southmoor Road, Manchester M23 9LT, UK

**Keywords:** histology, goblet cells, IgA, basement membrane, immune cells, collagen

## Abstract

**Background**: Airway remodeling is a cardinal feature of chronic obstructive pulmonary disease (COPD) pathology. However, inconsistent findings have been reported regarding the nature of proximal airway remodeling in COPD. This is likely due to the heterogeneity of COPD. This study investigated the histopathological features of airway remodeling in bronchial biopsies of COPD patients compared to smoking controls (S). We tested the hypothesis that histopathological features in bronchial biopsies relate to clinical characteristics in COPD patients, focusing on smoking status, symptom burden, lung function, exacerbation risk and inhaled corticosteroid (ICS) use. **Methods**: We recruited 24 COPD patients and 10 S. We focused on reticular basement membrane thickness (RBM), surface immunoglobulin A (IgA) expression, goblet cell numbers (periodic acid-Schiff [PAS]+), sub-mucosal remodeling markers including collagen 4, 6 and laminin expression, and inflammatory cell counts (CD45+). **Results**: RBM thickness was increased in frequent exacerbators, IgA expression was reduced in COPD patients with worse lung function, and goblet cell numbers were increased in COPD patients compared to S but with no difference between the COPD subgroups. Collagen 4 expression was associated with higher symptom burden and worse quality of life. Sub-mucosal inflammatory cell counts were increased in COPD non-inhaled corticosteroid (ICS) users compared to ICS users and S. **Conclusion**: We observed relationships between the histopathological features of airway remodeling and clinical characteristics in COPD patients. Our data highlight the influence of clinical heterogeneity on diverse patterns of airway remodeling in COPD patients.

## 1. Introduction

Chronic obstructive pulmonary disease (COPD) is caused by exposure to inhaled noxious particles, most commonly from cigarette smoking [1]. Persistent airway inflammation is a characteristic feature of COPD, involving various cell types including macrophages, neutrophils, and lymphocytes [2]. Chronic airway inflammation results in lung tissue injury, remodeling and, ultimately, destruction [3].

Airway remodeling in COPD affects both the proximal and peripheral airways. Previous histopathology studies using bronchial biopsies have shown increased basement membrane thickness, collagen deposition and goblet cell hyperplasia in COPD patients compared to smoking controls [4,5,6]. However, these findings are not consistent across studies, with no difference between groups or the reverse findings also reported [7,8,9,10]. A likely explanation for the variability between studies is the heterogeneity of COPD, as the clinical and pathophysiological features vary considerably between individuals. The clinical heterogeneity of COPD includes current smoking status (i.e., current, or ex-smokers), symptom severity, and the degree of lung function impairment. Exacerbations, defined as acute worsening of respiratory symptoms requiring additional therapy [1], are also heterogeneous, as some COPD patients experience frequent exacerbations (≥2 per year) whereas other patients experience none [11].

Some patients with COPD have increased susceptibility to bacterial infection, with reduced macrophage phagocytosis and peripheral airway immunoglobulin A (IgA) secretion identified as causative mechanisms [12,13]. Bacterial colonization can cause increased levels of neutrophilic airway inflammation [14], which may further promote tissue injury and repair processes.

This study investigated the histopathological features of airway remodeling in bronchial biopsies of COPD patients versus controls, focusing on reticular basement membrane thickness (RBM), surface IgA expression, goblet cell numbers, and sub-mucosal remodeling markers including collagen expression. The novelty of this study is that we performed detailed clinical phenotyping so that we could test the hypothesis that histopathological features in bronchial biopsies relate to clinical characteristics in COPD patients, namely smoking status, symptom burden, lung function, and exacerbation risk.

## 2. Methods

### 2.1. Study Subjects

24 COPD patients and 10 smoking controls (S) were recruited for bronchoscopy. This study was conducted in accordance with the Declaration of Helsinki 1975. The study was approved by South Manchester REC 06/Q1403/156 and all subjects provided written informed consent.

Demographic details were collected at the screening visit. All subjects had ≥10 pack years smoking history. Symptoms were assessed using the COPD assessment test (CAT). Health-related quality of life was measured using the St George’s Respiratory Questionnaire (SGRQ). Chronic bronchitis was determined using the American Thoracic Society (ATS) definition. Spirometry was performed in accordance with European Respiratory Society (ERS) and ATS guidelines [15]. COPD patients had a post-bronchodilator forced expiratory volume in 1 sec (FEV_1_) and forced vital capacity (FVC) ratio <0.7, with no history of asthma. We only recruited patients with no exacerbations (NoE) in the previous year and frequent exacerbators (FE) who experienced 2 or more exacerbations in the previous year.

### 2.2. Endobronchial Biopsy Collection and Immunohistochemistry

Endobronchial biopsies were collected from airway generations 2–5 using 2 mm radial jaw biopsy forceps (Boston Scientific, Hemel Hempstead, UK) and immediately fixed in 4% neutral buffered formalin, before being processed and embedded in paraffin wax. The biopsies were cut into 3 μm sections using a Leica RM microtome and lifted onto positively-charged adhesive glass slides (X-tra^®^, Leica Biosystems, Milton Keynes, UK). The sections were stained with hematoxylin and eosin to quantify RBM thickness. Goblet cells were quantified using periodic acid-Schiff (PAS). Immunohistochemistry assessed IgA2 (clone-RM125 Abcam, Cambridge, UK) and CD45 (clone-2B11 Dako, Stockport, UK) expression using ImmPRESS secondary reagent (VectorLabs, Peterborough, UK) and immunofluorescence assessed collagen 4, 6 and laminin expression. Further details can be found in the online supplement.

The bronchial biopsies were imaged using a Nikon Eclipse 80i microscope (Nikon UK Ltd., Milton Keynes, UK) equipped with a MicroPublisher 6 digital camera (QImaging, Birmingham, UK), and analyzed using ImagePro Plus-6.0. PAS^+^ cells were quantified within the epithelium, CD45^+^ cells were quantified within the epithelium and lamina propria (maximum distance of 125 µM from BM). IgA2 expression was expressed as the area of immunoreactive epithelium. Collagen 4, 6 and laminin expression were analyzed by colour deconvolution. Full details can be found in the online supplement. The analysis was conducted blind by one observer. Ten percent of samples were randomly selected for quality control by a second observer, where a coefficient of variation of <10% was deemed acceptable.

### 2.3. Statistical Analysis

No formal power calculation was performed; the sample size was based on previous studies with bronchoscopy sampling [8,9]. Statistical analyses were performed using GraphPad InStat software (GraphPad Software Inc, La Jolla, CA, USA). Data distributions were determined by the D’Agostino and Pearson normality test. Comparisons between two groups were made using an unpaired *t*-test or Mann–Whitney test. Comparisons between three groups were made using one-way ANOVA followed by Tukey’s test or Kruskal–Wallis followed by Dunn’s test. Pearson or Spearman correlations were performed to determine associations between histopathological measurements and clinical measurements.

## 3. Results

### 3.1. Study Subjects

The clinical characteristics of the study population are presented in Table 1. In the COPD group, the mean age was 64 years, slightly older than S (mean 53 years). For the COPD patients, the mean FEV_1_% predicted was 57%, 10/24 were current smokers (42%), 19 used inhaled corticosteroids (ICS), 10 had chronic bronchitis and the mean scores for the St George’s Respiratory Questionnaire (SGRQ) and COPD Assessment Test (CAT) were 37 and 16, respectively.

### 3.2. Reticular Basement Membrane

**Figure 1:**
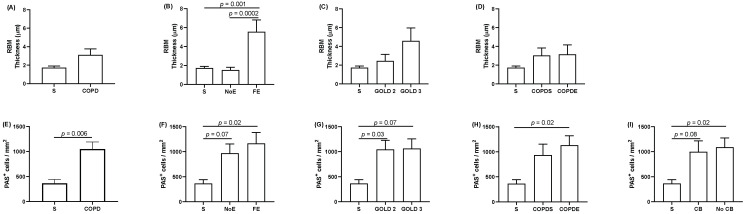
Quantification of reticular basement membrane thickness and goblet cells. The reticular basement membrane (RBM) thickness (**A**–**D**) and goblet cells (**E**–**I**) were compared between (**A**,**E**) COPD patients and smoking controls (S), with further detailed clinical phenotyping in COPD patients based upon (**B**,**F**) exacerbations, (**C**,**G**) airflow limitation, (**D**,**H**) smoking status, and (**I**) chronic bronchitis. Data presented as mean + SEM. Comparisons between groups were made by one-way ANOVA followed by Tukey’s test.

There was no significant difference in the RBM thickness between COPD patients and S despite a numerical trend for increased thickness in COPD (*p* = 0.2; Figure 1). The RBM thickness was significantly higher in FE compared to S and NoE (*p* = 0.001 and *p* = 0.0002 respectively; Figure 1). There was a non-significant trend for increased RBM thickness in GOLD 3 patients (ANOVA *p* = 0.08; Figure 1). There was no significant difference when COPD patients were separated by smoking status or ICS use (ANOVA *p* = 0.4 and *p* = 0.1, respectively; Figure 1 and Supplementary Figure S1). There were no correlations between RBM thickness and either SGRQ or CAT scores (Table 2).

### 3.3. Mucosal Measurements

#### Goblet Cells

**Figure 2:**
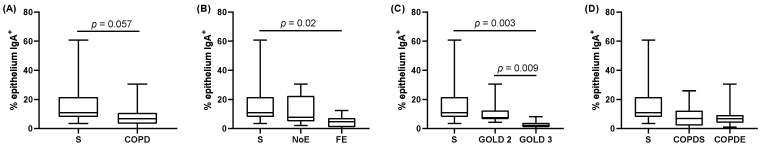
Quantification of IgA expression. IgA expression was compared between (**A**) COPD patients and smoking controls (S), with further detailed clinical phenotyping in COPD patients based upon (**B**) exacerbations, (**C**) airflow limitation, and (**D**) smoking status. Data presented as median ± range. Comparisons between groups were made by one-way Kruskal–Wallis followed by Dunn’s multiple comparison tests.

The number of PAS^+^ cells was significantly higher in COPD patients compared to S (*p* = 0.006 Figure 2). Similarly, when COPD patients were separated by exacerbation frequency, GOLD stage, smoking status, ICS use and chronic bronchitis, the number of PAS^+^ cells were higher compared to S (*p* = 0.02–0.1), but there were no differences between the COPD groups (Figure 2 and Supplementary Figure S1). The number of PAS^+^ cells were negatively correlated with SGRQ and CAT scores (rho= −0.4 *p* = 0.03 and rho= −0.4 *p* = 0.07, respectively) (Table 2). Because of the age difference between the COPD patients and the controls, we also performed a correlation between age and goblet cell numbers in COPD patients. This analysis confirmed age did not impact the results (rho = 0.3 and *p* = 0.2).

### 3.4. Surface Immunoglobulin A Expression

IgA2 expression was numerically lower in COPD patients compared to S, without reaching significance (*p* = 0.057, Figure 2). IgA2 expression was significantly lower in FE vs. S (*p* = 0.02, Figure 2), while the difference between FE and NoE was not significant (*p* = 0.1). IgA2 expression was significantly lower in GOLD 3 patients compared to S and GOLD 2 patients (*p* = 0.003 and *p* = 0.009, respectively, Figure 2). There was no difference between COPD patients grouped by smoking status or ICS use (Kruskal–Wallis *p* = 0.2 for both Figure 2 and Supplementary Figure S1). There were no correlations between IgA expression vs. SGRQ or CAT scores (Table 2).

### 3.5. Sub-Mucosal Measurements

**Figure 3:**
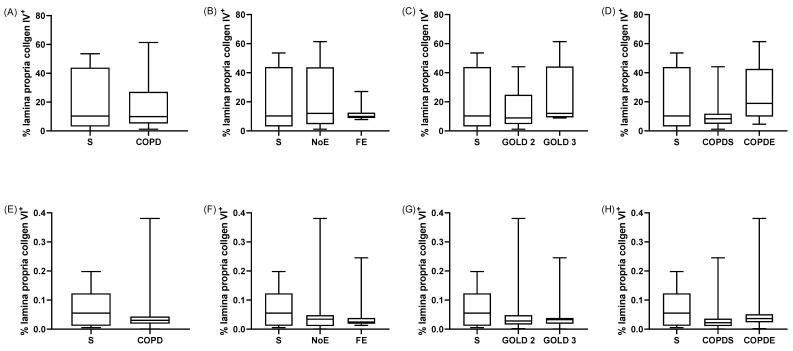
Quantification of collagen 4 and 6 expression. Collagen 4 (**A**–**D**) and 6 expression (**E**–**H**) were compared between (**A**,**E**) COPD patients and smoking controls (S), with further detailed clinical phenotyping in COPD patients based upon (**B**,**F**) exacerbations, (**C**,**G**) airflow limitation, and (**D**,**H**) smoking status. Data presented as median ± range. Comparisons between groups were made by Kruskal–Wallis followed by Dunn’s tests.

The percentage of lamina propria expressing collagen 4 was not different between COPD patients and S (*p* = 1.0, Figure 3). There were no differences between COPD patients when grouped by exacerbation frequency, GOLD stage, smoking status, and ICS use (*p* > 0.05, Figure 3 and Supplementary Figure S2). However, the percentage of lamina propria expressing collagen 4 was significantly correlated with SGRQ and CAT scores (rho = 0.7 *p* = 0.005 and rho = 0.6 *p* = 0.03, respectively).

**Figure 4:**
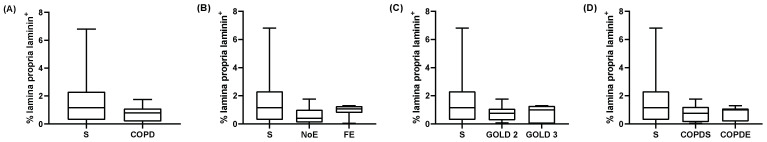
Quantification of laminin expression. Laminin expression was compared between (**A**) COPD patients and smoking controls (S), with further detailed clinical phenotyping in COPD patients based upon (**B**) exacerbations, (**C**) airflow limitation, and (**D**) smoking status. Data presented as median ± range. Comparisons between groups were made by one-way Kruskal–Wallis followed by Dunn’s tests.

There were no differences between groups for the percentage of lamina propria expressing collagen 6 or laminin when different groups were compared (Figure 3 and Figure 4, Supplementary Figure S2 and Table S2). Further information is provided in the online supplement.

### 3.6. Inflammatory Cell Measurements

**Figure 5:**
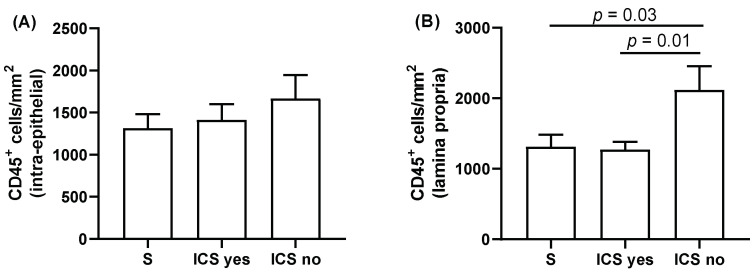
Quantification of airway immune cell counts in ICS users and non-users. Intra-epithelial (**A**) and sub-mucosal (**B**) CD45^+^ immune cells numbers were compared between ICS users (ICS yes), ICS non-users (ICS no), and smoking controls (S). Data presented as mean + SEM. Comparisons between groups were made by one-way ANOVA followed by Tukey’s multiple comparison test.

The number of CD45^+^ cells in the epithelium and lamina propria, were similar between COPD patients and S, and when COPD patients were grouped by exacerbation frequency, GOLD stage and smoking status (*p* = 0.1–0.7 Supplementary Figure S3). However, the number of CD45^+^ cells in the lamina propria was significantly higher in ICS non-users compared to S and ICS users (*p* = 0.03 and *p* = 0.01, respectively, Figure 5), with no difference between the groups for CD45^+^ cells in the epithelium when analyzed according to ICS use. There were no correlations between the number of CD45^+^ cells and SGRQ and CAT scores (Table 2).

## 4. Discussion

This study demonstrates the associations between histopathological features of airway remodeling in bronchial biopsies and clinical characteristics in COPD patients. The key findings were that RBM thickness was increased in frequent exacerbators, and that mucosal IgA expression was reduced in COPD patients with worse lung function. Goblet cell numbers were increased in COPD patients compared to S but not different between COPD subgroups defined by clinical characteristics. The findings for sub-mucosal remodeling markers were negative for between group comparisons, although collagen 4 expression was associated with a higher symptom burden and worse quality of life. We also observed that sub-mucosal inflammatory cell counts were increased in COPD non-ICS users compared to COPD ICS users and S.

The RBM provides structural support to the airways, with its structure influenced by physiological signals from the overlying airway epithelium and underlying mesenchyme [16]. Thickening of the RBM is a recognized feature of airway remodeling in asthma, but the evidence from COPD studies is mixed [6,9,10]. We hypothesized that previous conflicting results could be due to COPD heterogeneity. Our results support our hypothesis, as we observed clearly increased RBM thickening in the COPD subgroup with frequent exacerbations. This implicates exacerbation events as potential drivers of RBM thickening, potentially due to airway inflammation and consequent tissue injury during exacerbations [17]. However, we have described associations but not a direction of causality. Nevertheless, the transmigration of immune cells through the RBM can cause RBM degradation which requires repair [18], suggesting a mechanism by which excessive RBM thickening occurs as a consequence of frequent exacerbations.

IgA is an integral component of anti-microbial mucosal defense [19]. It has previously been shown that COPD patients with worse lung function have lower surface IgA in the small airways [20]. We now demonstrate similar findings in COPD large airways, with lower surface IgA associated with worse lung function. Reduced IgA expression in COPD small airways is associated with increased bacterial colonization, while lower serum IgA is associated with increased COPD exacerbation risk [13,21]. These previous findings, considered alongside our current results, indicate that increasing COPD severity (determined by FEV_1_) is associated with reduced anti-bacterial mucosal defense in the large and small airways, predisposing to bacterial colonization. Future studies should investigate the relationship between mucosal and serum IgA expression to examine the utility of serum IgA as a prognostic marker of disease manifestation.

Goblet cell hyperplasia is a common feature of small airway remodeling in COPD but evidence in large airway studies is mixed [3,4,8,22,23]. Whereas Innes et al. showed increased goblet cell numbers were associated with greater airflow obstruction [4], Kim et al. showed the reverse to be true [8]. Moreover, increased goblet cell numbers have been linked to smoking status, but the relationship with chronic bronchitis is less clear, regardless of airflow limitation [8,23]. In our study, we did not observe a relationship to either of these clinical characteristics, although there was a clear increase in COPD patients compared to smoking controls. Perhaps paradoxically, we observed an inverse relationship between goblet cell numbers and COPD symptoms (measured by CAT score) and quality of life, which at first glance may seem somewhat surprising due to the link between goblet cell physiology and mucous production, particularly mucous plugging of the peripheral airways [2]. However, animal model data demonstrates that MUC5B deficient mice exposed to cigarette smoke have reduced goblet cell numbers and increased airway inflammation compared to wildtype mice, suggesting a protective role for goblet cells during cigarette smoke exposure [24]. Perhaps our data demonstrates a distinction between proximal and peripheral airways; in the large airways examined here, increased mucous production may remove pathogens and, thereby, regulate inflammation. Increased goblet cell numbers in the large airways may, therefore, be protective and provide a potential explanation for the relationship with symptoms and quality of life observed.

The extracellular matrix is a complex mixture of macromolecules including collagens and proteoglycans, the proportion of which determines airway mechanics [25]. Increased collagen 6 and laminin, but not collagen 4 expression, have been observed in the large airways of COPD patients compared to controls [5,7,26]. These proteins are not only important for airway structure but can also influence immune cell activation and microbial adhesion [26,27,28]. We did not observe changes to collagen 4, 6 and laminin expression between groups. The reason for this lack of difference for collagen 6 and laminin is unclear, in the context of between group differences previously described [5,26]. Perhaps dynamic changes in extracellular matrix (ECM) turnover is important here; for example, breakdown products of collagen 6 increase during COPD exacerbations and then decrease over time [29]. Another possibility is that the expression of these proteins is patchy, rather than uniform, throughout the large airways. However, we observed increased collagen 4 expression only is related to increased symptom burden in COPD patients, although not lung function. Collagen 4 is a non-fibrillar collagen that functions to join fibrillar collagens and other scaffold proteins together to provide the structural integrity of the ECM [30]. Increased collagen deposition has been linked to a fibrotic remodeling process that increases tissue stiffness [31]. Whether increased collagen 4 deposition increases airway stiffness in COPD is yet undetermined, but this could explain, mechanistically, the relationship to increased symptom burden in our study.

It is generally accepted that the number of sub-mucosal immune cells are increased in the large airways of COPD patients compared to controls. However, there are inconsistencies within these studies. For example, whereas O’Shaughnessy et al. showed increased numbers of CD3^+^ CD4^+^ lymphocytes in COPD versus controls, Lams et al. and Battaglia et al. found no differences between groups [32,33,34]. Similar observations have also been reported for macrophages and neutrophils [32,33,34,35]. However, these studies did not control for ICS use, which may be a confounding factor, as observed in randomized controlled trials that have shown reduced sub-mucosal inflammatory cells counts with ICS treatment when combined with a long-acting beta agonist [36,37]. We observed that the numbers of immune cells (CD45^+^) are increased in COPD patients who do not use ICS compared to both smoking controls and COPD patients using ICS; confirming that ICS reduce immune cell infiltration in COPD patients.

Our study has limitations. We performed multiple comparisons, increasing the chances of false positive findings with limited sample sizes in subgroups. However, some of our results match previous data, notably that reduced IgA expression is related to disease severity and that goblet cell hyperplasia is increased in COPD compared to controls [4,20], providing confidence that the sample sizes were sufficient. Another limitation is that we did not stain for individual immune cells; rather, we used a pan immune cell marker. In addition, we cannot definitively ascertain the cause and effect relationship between the histopathological features of airway remodeling and clinical characteristics.

In conclusion, we have demonstrated relationships between histopathological features of airway remodeling and clinical characteristics in COPD patients. These data highlight the influence of clinical heterogeneity on diverse patterns of airway remodeling in COPD patients.

## Figures and Tables

**Table 1:** Clinical characteristics of the study population.

	Smoking Controls	COPD	*p*-Value
*n*	10	24	
Age (years)	53 ± 8	64 ± 6	<0.0001
Gender: % male	50	83	0.04
Current smoker %	100	42	0.002
Pack years	29 ± 15	43 ± 23	0.09
Exacerbation group *n* (%)			
NoE	*n*/a	14 (58)	*n*/a
FE		10 (42)	
FEV_1_ (L)	2.9 ± 0.7	1.6 ± 0.5	<0.001
FEV_1_ (%)	97 ± 14	57 ± 11	<0.001
FEV_1_/FVC ratio (%)	76 ± 4	44 ± 9	<0.001
GOLD category *n* (%)			
1	*n*/a	0	*n*/a
2		17 (71)	
3		7 (29)	
4		0	
SGRQ	*n*/a	37 ± 26	*n*/a
CAT	*n*/a	16 ± 11	*n*/a
Chronic Bronchitis (%)	*n*/a	42	*n*/a
ICS users (%)	*n*/a	79	*n*/a

Data are presented as mean ± SD. NoE no exacerbations; FE frequent exacerbators; FEV_1_ forced expiratory volume in one second; FVC forced vital capacity; ICS inhaled corticosteroids; CAT COPD Assessment Test; SGRQ St George’s Research Questionnaire; n/a not applicable.

**Table 2:** Relationship between histopathological measurements and symptom scores.

	SGRQ Score	CAT Score
RBM thickness	Rho= −0.1 *p* = 0.8	Rho= −0.02 *p* = 0.9
% epithelium IgA2^+^	Rho= −0.2 *p* = 0.3	Rho= −0.3 *p* = 0.2
PAS^+^ cells/mm^2^	Rho= −0.4 *p* = 0.03	Rho= −0.4 *p* = 0.07
% LP expressing collagen 4	Rho = 0.7 *p* = 0.005	Rho = 0.6 *p* = 0.03
% LP expressing collagen 6	Rho= −0.1 *p* = 0.7	Rho= −0.1 *p* = 0.8
% LP expressing laminin	Rho= −0.4 *p* = 0.1	Rho= −0.3 *p* = 0.2
CD45^+^ cells/mm^2^ intra-epithelium	Rho= −0.1 *p* = 0.7	Rho= −0.03 *p* = 0.9
CD45^+^ cells/mm^2^ LP	Rho= −0.3 *p* = 0.2	Rho= −0.3 *p* = 0.2

## Data Availability

The datasets generated and/or analysed during the current study are not publicly available.

## Supplementary Materials

The following supporting information can be downloaded at: https://www.mdpi.com/article/10.3390/biomedicines10081992/s1, Figure S1: Histopathological measurements in ICS users vs. non-users; Figure S2: Histopathological measurements in ICS users vs. non-users; sub-mucosal; Figure S3: Quantification of airway immune cell counts.
