# Supplementary figures and images for: Relationships between Airway Remodeling and Clinical Characteristics in COPD Patients
Source: Biomedicines. 2022 Aug 17;10(8):1992. doi: 10.3390/biomedicines10081992 (PMC9405811; doi:10.3390/biomedicines10081992)

SUPPLEMENTARY FIGURE S1

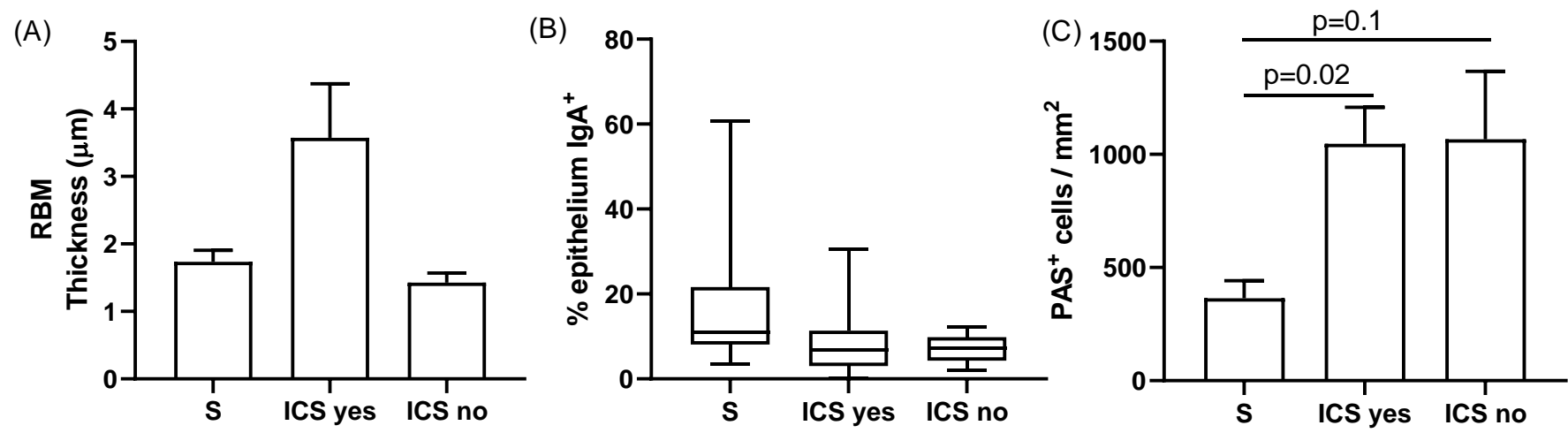

SUPPLEMENTARY FIGURE S2

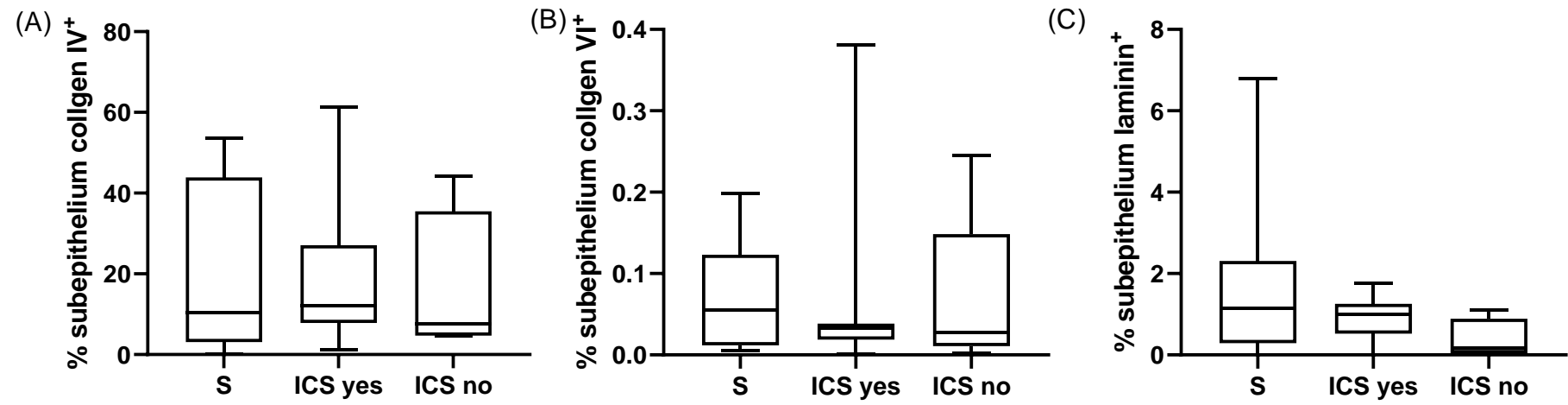

SUPPLEMENTARY FIGURE S3

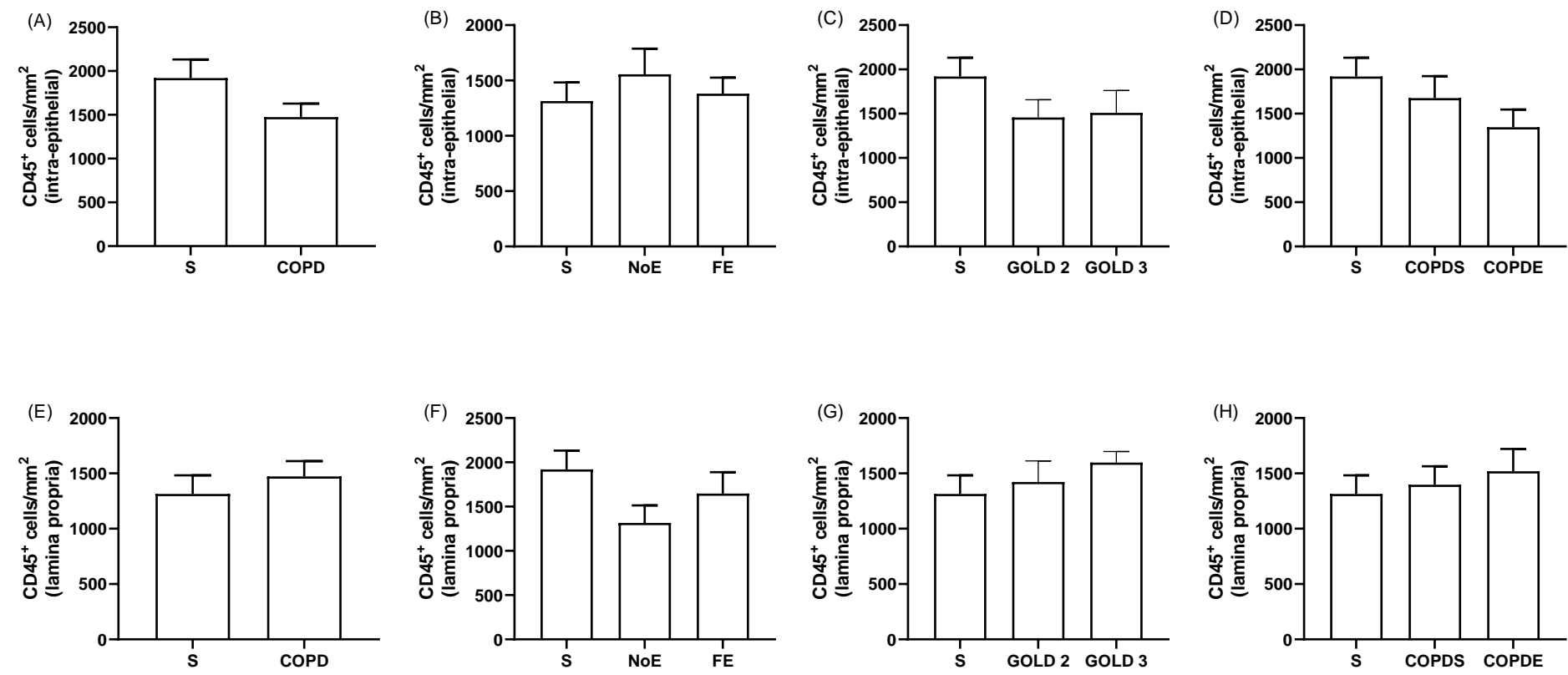

Supplement: Supplementary file 1 [file biomedicines-10-01992-s001.zip › biomedicines-1821906-supplementary.pdf]
